# Using Translational Models of Fear Conditioning to Uncover Sex-Linked Factors Related to PTSD Risk

**DOI:** 10.20900/jpbs.20220010

**Published:** 2022-11-07

**Authors:** Anna M. Rosenhauer, Brittney Owens, Ebony M. Glover

**Affiliations:** 1 Department of Psychological Sciences, Kennesaw State University, Kennesaw, GA 30144, USA; 2 Department of Molecular and Cellular Biology, Kennesaw State University, Kennesaw, GA 30144, USA

**Keywords:** fear conditioning, gonadal hormones, PTSD, sex differences, fear learning, extinction, fear-potentiated startle, skin conductance response

## Abstract

Post-traumatic stress disorder (PTSD) is a debilitating neuropsychiatric disorder that follows exposure to a traumatic event; however, not every person who experiences trauma will develop PTSD. Women are more likely to be diagnosed with PTSD than men even when controlling for type and amount of trauma exposure. Circulating levels of gonadal hormones such as estradiol, progesterone, and testosterone may contribute to differential risk for developing PTSD. In this review, we briefly consider the influence of gonadal hormones on fear conditioning processes including fear acquisition, fear inhibition, extinction learning, and extinction recall within translational neuroscience models. We discuss findings from human studies incorporating samples from both community and traumatized clinical populations to further understand how these hormones might interact with exposure to trauma. Additionally, we propose that special attention should be paid to the specific measure used to examine fear conditioning processes as there is evidence that common psychophysiological indices such as skin conductance response and fear-potentiated startle can reveal quite different results and thus necessitate nuanced interpretations. Continued research to understand the influence of gonadal hormones in fear learning and extinction processes will provide further insight into the increased risk women have of developing PTSD and provide new targets for the treatment and prevention of this disorder.

## INTRODUCTION

Post-traumatic stress disorder is a debilitating disorder that follows exposure to a traumatic event. Although, as much as 90% of the US population is exposed to at least one traumatic event during their lifetime [1], only one-tenth of those who survive life-threatening events will develop PTSD [2]. Differential risk for developing PTSD is multidetermined but in part depends on sex, with women having approximately twice the risk as men of developing PTSD following trauma exposure [1–3]. Women also tend to have a four-fold longer course of illness and are more likely to utilize urgent care services, which come with a higher cost burden [1,4–6]. Even after controlling for trauma exposure factors, sex differences in PTSD remain [1–3]; thus, it is necessary to understand biological factors contributing to female’s increased risk for PTSD. This review will consider hormonal evidence from translational models of fear conditioning in humans, which model clinical presentations of PTSD symptoms and treatment. We will overview the contribution of gonadal hormones to women’s risk of developing PTSD and the importance of considering specificity of measurement to ensure that contributions of hormones are not inadvertently overlooked.

PTSD symptoms are characterized by exaggerated fear and anxiety behaviors triggered by learned environmental cues that serve as reminders of a prior traumatic experience [7]. Preclinical research aimed at understanding factors that regulate fear learning and memory processes in high-risk populations could shed light on the biological underpinnings of PTSD risk and resilience. Clinical presentations of PTSD symptoms can be modeled in the laboratory via differential fear conditioning in which neutral stimuli are contemporaneously paired with or without an aversive stimulus (unconditioned stimulus or US). The neutral stimulus paired with threat (conditioned stimulus or CS+) gains emotional significance and begins to trigger fear and arousal response as it serves as a reminder of threat. The other neutral stimulus (CS−) becomes a reminder cue for safety and signals the inhibition or suppression of threat responding. Fear extinction is another laboratory model for testing fear inhibition. For fear extinction, the CS+ that was paired with an aversive stimulus during acquisition is repeatedly presented without the aversive stimulus, thus allowing the fear response to extinguish [8]. While fear acquisition refers to learning that something is dangerous, extinction is a mechanism by which an individual learns that the stimulus no longer represents threat. This procedure forms the theoretical basis for cognitive behavioral or exposure therapy, the first-line treatment for PTSD. Impaired fear extinction is widely considered a key component of PTSD [9–11]. Extinction learning and memory can be further examined by retesting participants after the passage of time to determine the strength of extinction retention or whether conditioned fear expression will reemerge —a phenomenon termed fear recovery. Extinction recall has been shown to be deficient in PTSD [12]. Understanding the processes by which fear extinction is retained has the potential to inform treatments for PTSD that lead to lasting changes in fear responses.

Despite the increased risk of females developing PTSD, limited studies have investigated the role of ovarian hormones using translational models of fear conditioning. Brain regions involved in fear learning and memory processes such as the amygdala, prefrontal cortex, and hippocampus [13] show structural, cellular, and molecular differences based on sex [14]. These brain regions contain elevated levels of ovarian hormone receptors [15], thus, the action of ovarian hormones in these brain regions may explain in part, or contribute to, the increased risk of women to developing PTSD. Ovarian hormones including estradiol and progesterone fluctuate throughout the menstrual cycle. Unfortunately, a substantial number of translational fear conditioning studies have failed to account for cycle phase or measure ovarian hormones, potentially overlooking a significant source of variation in responses to fear and extinction learning in females, and possibly neglecting sources of potential increased risk for females. This review will consider studies investigating gonadal hormones’ effects on fear conditioning processes in humans and discuss potential future directions for studies that could help uncover the biological mechanisms underlying women’s increased risk for developing PTSD.

## GONADAL HORMONE INFLUENCES ON FEAR LEARNING AND MEMORY

**Figure 1.**
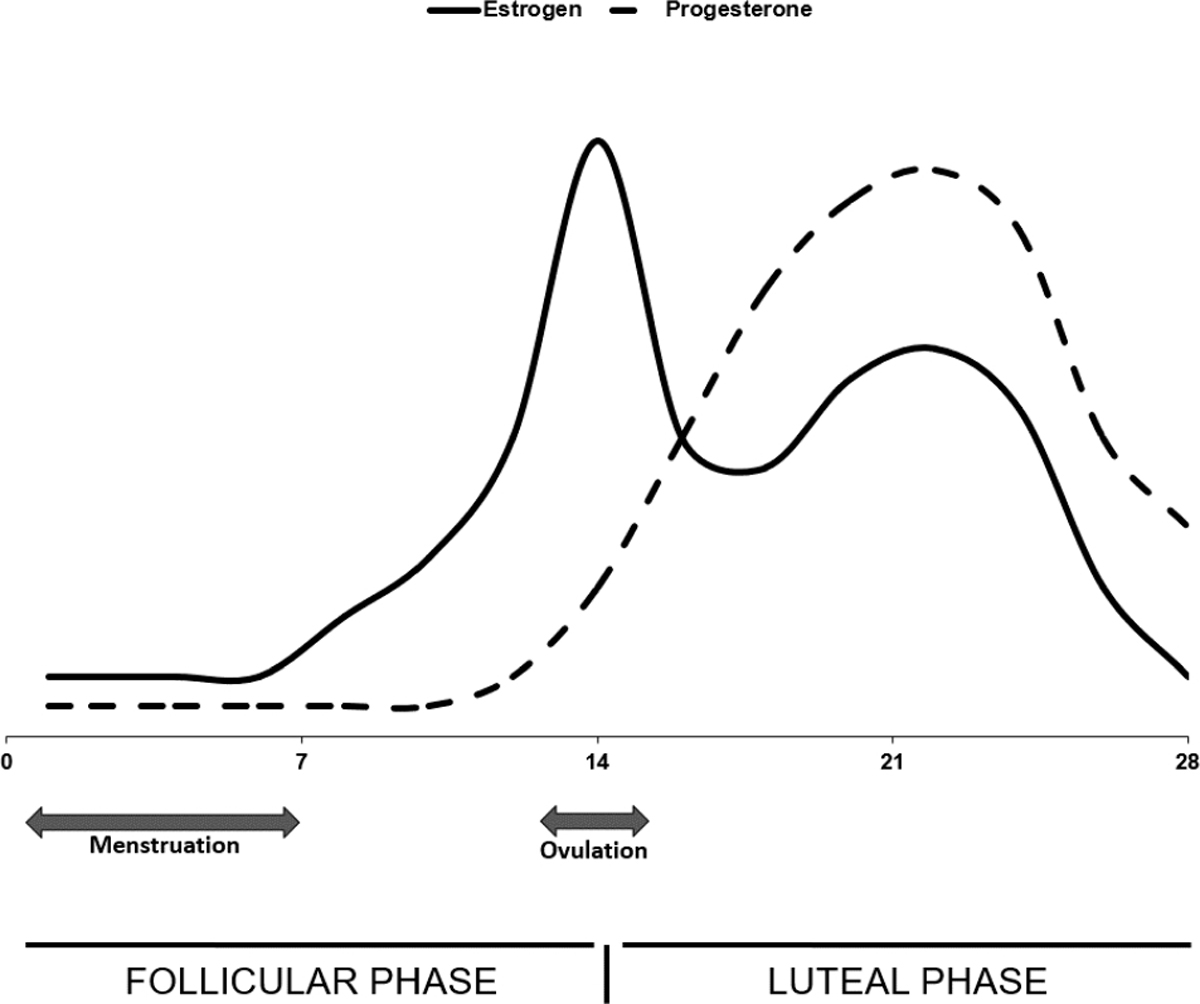
Typical estrogen and progesterone fluctuations across the 28-day human menstrual cycle. The solid line represents estrogen levels, which peak at ovulation and remain relatively elevated during the luteal phase. The dashed line represents progesterone level, which peaks after ovulation and is high during the luteal phase (adapted from [16] copyright © CMAJ Group).

Estrogens are the primary female sex hormones, with estradiol being the most potent in nonpregnant females. During the menstrual cycle, estradiol is lowest immediately before and during the time of menstruation and has its first peak during the late-follicular phase with a second, lower peak that roughly corresponds with the peak of progesterone during the mid-luteal phase (see Figure 1).

There is little evidence that estradiol impacts fear acquisition in animal models, but some suggestion that there is faster extinction of contextual fear conditioning during periods of high estradiol levels and with exogenous administration of estradiol to ovariectomized animals; however, there is little evidence that estradiol impacts extinction of cued fear. In rodents, considering estrous cycle phase results in extinction recall differences in females with extinction being facilitated during days when estrogen and progesterone are higher [17]. Also, estradiol or progesterone administration resulted in enhanced extinction recall [17,18] with possible interactions between estradiol and progesterone [19]. The animal research is sparser for progesterone alone. Progesterone was shown to enhance cued memory in aged mice [20]. Testosterone is another gonadal hormone, more plentiful in males than in females. Animal studies are not conclusive on the role of testosterone in fear conditioning. Some research found no effect of gonadectomy on hippocampal contextual fear memory [21]. On the other hand, multiple lines of evidence support testosterone’s role in increasing contextual fear memory without impacting amygdalar cued fear memory [22,23]. Intriguingly, in one study testosterone decreased cued fear memory in male mice, but not females [24]. Overall, in animal studies, the most conclusive evidence points to estradiol’s impact on extinction recall; however, fewer studies have examined the role of estradiol in fear conditioning processes in humans.

## GONADAL HORMONE EFFECTS IN COMMUNITY SAMPLES

Two different populations have been investigated in humans using translational models of fear conditioning to examine ovarian hormones with regard to PTSD. Community samples consist primarily of individuals who are free from major neuropsychiatric disorders, with no or limited individuals with PTSD. Clinical samples have also been investigated with individuals who are highly traumatized and who do or do not meet the criteria for a PTSD diagnosis. Community samples have primarily used skin conductance response and expectancy ratings to investigate fear acquisition, extinction, and extinction recall. Of these studies, only a few in humans have reported differential responding based on estradiol levels during fear acquisition. In one, participants with spider phobia with low estradiol levels demonstrated increased skin conductance response (SCR) to the danger signal (CS+) compared to other women during fear acquisition [25]. Felmingham and colleagues found no significant effect of estradiol levels on acquisition, but participants with higher estradiol did have decreased threat expectancy ratings for the safety cue during acquisition. This suggests a possible route for a protective effect of estradiol on cognitive discrimination between cues [26]. Graham and colleagues also found no differences in acquisition based on hormone level but did find that high estradiol was associated with decreased SCR and lower expectancy ratings in response to the danger signal following instruction in cognitive restructuring [27]. Indeed, in the second fear acquisition phase after cognitive restructuring, higher levels of serum estradiol were associated with lower CS+ elicited SCRs. Although working with a small clinical sample, Levy et al., found that women with anxiety disorders showed no SCR differences in SCR based on estradiol levels following cognitive restructuring that more closely resembled clinical practice, indicating that the impact of estradiol might be specific to basic fear conditioning processes and may not translate to clinical practices [28]. Taken together, there seems to be some evidence that under specific conditions (i.e., phobia or following cognitive restructuring) low levels of estradiol might increase response to the danger signal and that higher levels of estradiol are associated with decreased responding to the danger signal during fear acquisition. At least in the case of estradiol’s impact following cognitive restructuring though, this finding may not translate directly to more clinical models.

When investigating fear extinction, Bierwirth et al. found that men displayed greater SCR during extinction than women using hormonal contraceptives (low levels of estradiol and progesterone) and naturally cycling women tested during a high estradiol portion of the menstrual cycle (Bierwirth 2021)[29]. In contrast, two other studies found no impact of hormonal differences in fear extinction learning on SCR [25,30]. This discrepancy could be because the former study used male faces as stimuli, and there is some evidence that at least in youth, males show greater fear acquisition and decreased fear extinction learning in response to male faces [31]. Li and Graham did however show that women with high estradiol displayed greater CS− expectancies during early extinction trials, and hormonal contraceptive users displayed lower expectancy ratings to the safety signal than did men during fear extinction [32]. This sparse evidence might indicate a lack of hormonal influence on extinction learning processes at least when measured with SCR to traditional stimuli with some evidence that hormonal status could influence expectancy ratings in response to the safety signal (CS−) during extinction. Further studies with both traditional and non-traditional stimuli are needed to clarify this apparent contradiction.

The investigation of hormonal influences on extinction recall are the most plentiful, perhaps owing to positive results found in early studies. Multiple studies have found that higher levels of estradiol are associated with enhanced extinction recall when measured by SCR while lower levels of estradiol tend toward a greater recovery of the conditioned fear [25,29,30,32–34]; although one study demonstrated this only following a prior stressor [35]. A meta-analysis that considered multiple studies also confirmed this general result [36]. There is also evidence for a correlation between estradiol levels and fear recovery with low estradiol being associated with increased fear recovery (decreased extinction recall) [32]. Interestingly, when women’s reproductive status was divided by whether they had children or not, the association between estradiol and fear extinction recall was only seen in women without children [37]. In addition, Graham and Milad [30] demonstrated that exogenous estradiol administration was able to rescue extinction recall deficits in participants with lower levels of estradiol. Similarly, women who were given exogenous estradiol prior to fear extinction displayed enhanced extinction recall when compared to women who received only placebo [38]. However, not all results agree with these findings. Some studies have found that low levels of estradiol, due to either cycle phase or the use of hormonal contraceptives, were associated with enhanced extinction recall relative to participants with higher levels of estradiol [39,40]. Several explanations exist for this apparent contradiction including the use of self-report of cycle phase, which is notoriously erroneous, and the comparison of early follicular versus late follicular phases which may not precisely target differences in estradiol levels due to individual variance and differences in cycle length. So, although there is evidence that high estradiol levels are associated with enhanced extinction recall, more investigation is needed to understand these contrasting results.

Franke et al. explored the role of estradiol on intrusions following fear acquisition that paired aversive or neutral film clips with shock [41]. Intrusive memories are a major symptom of PTSD and are thought to represent a lack of inhibition, possibly related to decreased encoding and consolidation of contextual information alongside the traumatic association. Among participants with high state anxiety, low estradiol levels during conditioning were associated with increased intrusions early in the week following conditioning, but fewer intrusions at the end of the week, when compared with those with higher levels of estradiol. This suggests a complex interaction between anxiety state, estradiol levels, and time. Additionally, lower estradiol levels during conditioning were associated with increased somatic intrusions of pain during the week following conditioning. Also, when participants were re-exposed to the film clips, low estradiol during the original pairings predicted more painful responses to stimuli that were not previously paired with pain. This study is unique in its additional focus on intrusions of somatic sensations (i.e., pain), which is important due to PTSD’s high comorbidity with chronic pain and because intrusive somatic memories are a highly distressing symptom of PTSD. An additional study focused on fear reinstatement, a protocol in which the aversive US is presented without any cues following the extinction procedure, also found increased SCR with lower levels of estradiol [26].

## GONADAL HORMONE EFFECTS IN CLINICAL SAMPLES

Clinical samples of individuals who either have PTSD or are highly traumatized have shown similar findings. Glover et al. [16] examined both a community sample and a highly traumatized clinical sample. In the community sample, women in the luteal phase (higher levels of hormones) displayed significant fear potentiated startle (FPS) discrimination between the danger and safety cues during fear acquisition and significant inhibition of fear when a safety signal was combined with a danger signal. Women in the follicular phase, with lower levels of hormones, displayed neither discrimination nor fear inhibition, despite displaying cognitive awareness of the contingency. In the highly traumatized sample, women in the high estradiol group displayed significant discrimination between the danger and safety signal and significant inhibition of fear in the presence of the safety signal. Women in the low estradiol group did not display either discrimination or inhibition when measured via FPS. A study by Bartholomew and colleagues utilized a trauma-exposed sample and found that women on hormonal contraceptives (low levels of ovarian hormones but higher synthetic hormones) demonstrated a faster time course of fear learning when compared to naturally cycling women who were in the early follicular phase of their menstrual cycle (low estradiol and progesterone) and men [42]. This study is unique in its comparison of women on hormonal contraceptives and women with low endogenous estradiol and suggest there may be some enhancements in fear acquisition specifically due to synthetic hormones.

One naturalistic way to examine the impact of hormones in females is to compare those who are pregnant (with higher levels of estrogen and progesterone) with those who are not pregnant. One study with a highly traumatized sample found that only women who were not pregnant displayed discrimination between the safety and danger signal via FPS measure [43]. This lack of discrimination was due to an increase in startle to the safety signal seen in women who were pregnant relative to that shown by non-pregnant women, and this increased startle was associated with increased levels of hypervigilance [43]. Indeed, FPS to the safety signal was also positively associated with hyperarousal symptoms in PTSD, but only in pregnant women (with elevated levels of hormones). The lack of discrimination seen in pregnant women was not due to lack of contingency awareness as expectancy data demonstrated clear learning of the association between the danger signal and the aversive stimulus [43]. Thus, there is some evidence pointing to deficits in discrimination during fear acquisition with lower levels of ovarian hormones in highly traumatized individuals. This lack of discrimination between the danger and safety signal could be related to lack of fear inhibition and increased startle response to the safety signal despite individuals showing clear cognitive awareness of the US/CS+ contingency.

With regard to fear extinction learning, one study compared highly traumatized women who either did or did not meet criteria for a PTSD diagnosis. Interestingly, differences in extinction learning between participants with and without PTSD were revealed only among those with low estradiol. There were no differences in extinction based on PTSD diagnosis among those with high estradiol [44]. The afore-mentioned Bartholomew study that compared trauma-exposed women on hormonal contraceptives and women in the follicular phase of the menstrual cycle extinguished the previously conditioned fear 72 h after acquisition and found that women on hormonal contraceptives displayed a faster time course of extinction learning suggesting that synthetic hormones from hormonal contraceptives might be impacting both acquisition and extinction learning [42]. The results with fear acquisition, inhibition, and extinction in clinical samples contrasts with the lack of evidence for any hormonal impact on fear acquisition and extinction seen in community samples. A crucial difference that needs to be considered, however, is the measurement of fear conditioning processes with FPS rather than solely SCR. The previous community samples all used SCR to measure fear acquisition and extinction while some of the clinical samples also measured FPS which could further illuminate the impact of hormones on these processes. Indeed, some researchers found differences based on hormone levels in fear extinction learning only when measuring FPS and not SCR [44]. Although the differences in fear extinction learning with hormonal contraceptive use was shown using SCR in the Bartholomew study, differences in the learning outcome of acquisition or extinction were not found. The differences found were in the time course of fear and extinction learning which could explain this discrepancy [42]. Future work examining fear acquisition, inhibition, and extinction learning using FPS in community samples is needed as well as analyses on the time course of learning alongside specific learning outcomes to determine if study differences are responsible for these contradictory results.

When looking at extinction recall with participants divided by PTSD diagnoses, participants with PTSD in the early follicular phase (low estradiol and progesterone) displayed greater extinction recall than those in the mid luteal phase (higher hormone levels) when measured with SCR [45]. In contrast, among participants without PTSD, those in the mid luteal phase showed greater extinction recall than those in the early follicular phase. For those with PTSD, higher progesterone was associated with decreased extinction recall, but for those without PTSD lower estradiol and progesterone were associated with decreased extinction recall [45]. Interestingly, a follow up study found that extinction recall was positively related to plasma allopregnanolone and pregnanolone but only in participants with PTSD and only during the mid-luteal phase (when estradiol and progesterone are high) not during the early follicular phase (when both are low). In addition, the ratio of allopregnanolone/pregnanolone to dehydroepiandrosterone (DHEA) levels was positively associated with extinction recall only during the midluteal phase, with some evidence that this was more so in the PTSD group than the non-PTSD trauma-control group [46]. In the sample comparing women on or off hormonal contraceptives, extinction recall was tested 1 week after extinction, thus this compared women on hormonal contraceptives (low estradiol and low progesterone) to women in the late follicular phase (high estradiol and low progesterone [42]. Researchers detected an overall effect of sex in that women who were in the late follicular phase displayed lower overall SCR than men, but there was no impact of hormonal contraceptive status on extinction recall itself. Hence, there appears to be differences in the impact of estradiol and progesterone based on PTSD diagnosis in several fear conditioning processes and a possible impact of synthetic hormones from hormonal contraceptives on these processes. In addition, differences in the levels of these hormones could be interacting with other gonadal hormones. Failing to consider or account for menstrual cycle phase and hormonal contraceptive use has the potential to obscure the impact of other gonadal hormones particularly in clinical or traumatized samples, and additional studies are needed to further parse out these effects.

## PROGESTERONE

Though less widely studied than estradiol, progesterone is another ovarian hormone that fluctuates with the menstrual cycle and is decreased with hormonal contraceptive usage. Most studies with community samples found no influence of progesterone on fear acquisition, extinction, or extinction recall [30,33,34]. One study found that higher progesterone facilitated increased intrusive memories following high valence film clips [47], but other studies failed to find equivalent results [48–50]. A meta-analysis including several primary articles found that high progesterone and the luteal phase of the menstrual cycle when progesterone is high were positively correlated with increased negative intrusive memories [36]. The afore-mentioned study by Pineles and colleagues [45] found decreased fear extinction recall with higher progesterone in individuals with PTSD, but the opposite in individuals without PTSD. Some researchers have argued that progesterone’s relationship to cortisol and brain-derived neurotrophic factor acting in concert with estradiol makes it a particularly likely candidate in vulnerability of women to PTSD [51]. It is difficult to dissociate the effects of progesterone and estradiol; however, because progesterone varies with estradiol, and this might obscure the impact of progesterone on fear conditioning processes. Some studies have used a ratio of progesterone and estradiol levels to better examine this, but results have been mixed. A median split with the P/E ratio found no differences in fear conditioning processes [34]. On the other hand, P/E ratio was negatively correlated with intrusive memories following film clip viewing [49] indicating that perhaps the P/E ratio might be important to consider when investigating PTSD-related symptoms such as re-experiencing and intrusions. In addition, it has been suggested that considering menstrual cycle phase might be necessary for appropriate use of the P/E ratio [52]. Overall, further research is needed to understand the interaction between progesterone and estradiol and how this in turn impacts fear conditioning in humans.

## TESTOSTERONE

Testosterone is another gonadal hormone with possible importance for elucidating sex differences in fear conditioning and PTSD. Testosterone has anxiolytic qualities [23,53,54]; however, its role specifically in fear learning and memory remains unclear. In humans, a single dose of testosterone given to females was shown to decrease fear-potentiated startle [55]. Similarly, testosterone attenuated a greater reduction of fear measured by the acoustic startle response due to the valance of images that was seen in women with high anxiety. When measuring fear using SCR, testosterone likewise attenuated the increase in SCR seen with negatively valanced images [56]. These studies indicate that testosterone decreases both the amygdala-based fear-potentiated startle and the general increase in arousal measured with SCR, possibly under specific circumstances such as negative valances or cues. Thus, while evidence is varied, the role of testosterone appears to differentiate between hippocampal-based contextual learning and amygdala-based cued learning with higher testosterone increasing expression of contextual fear but having no effect or even decreasing the expression of cued fear at recall at least in rodents [22,23]. Further studies are needed to better understand testosterone’s role in fear conditioning and its possible role in sex differences seen in PTSD diagnoses and symptomology.

## MEASUREMENT SPECIFICITY

The two most common indices of threat response in human studies are an increase in skin conductance response and acoustic startle amplitude [57]. Although both measure fear conditioning, it is important to understand their differences. Skin conductance response (SCR) which measures electrodermal activity is the most common psychophysiology measurement used in translational studies related to PTSD and fear conditioning. Studies most often use SCR as a proxy for fear as SCR is a reliable index of conditioned fear [58–61], but it primarily measures the autonomic nervous system’s activation and arousal. Importantly, SCR does not have a solid counterpart in studies using animal models. Thus, our understanding of its neural correlates is limited. The mammalian acoustic startle reflex, unlike skin conductance, is mediated by a direct neural pathway to the amygdala. [57,62,63]. Fear potentiated startle (FPS) is the heightened startle amplitude in response to an unpleasant conditioned stimulus most often measured via eyeblink electromyography [57,64,65]. FPS can be readily modeled in numerous species. In combination with its direct pathway to the amygdala, FPS could possibly produce fear responses not normally seen when utilizing SCR alone. The most precise evaluation of the role that hormones and biological sex play in PTSD should include all modalities of assessment. When using FPS or SCR to model an actual threat, researchers should practice caution when interpreting results and not form assumptions about the function of hormones in fear conditioning and PTSD without considering multiple measures. Fear responses unique to an individual are frequently missed by SCR testing alone, and this is true of individuals with and without PTSD [66]. Ultimately, FPS may be the better measure when looking for statistically significant relationships between hormones and biological sex differences in individuals with PTSD.

Some studies using both SCR and FPS to examine fear conditioning have demonstrated the double dissociation between these responses [67,68]. SCR is more closely related to arousal regardless of valence whereas FPS is a more direct index of fear [69]. One reason that SCR is more common than FPS in human fear conditioning studies is the ease with which SCR can be measured within fMRI paradigms. The easiest measurement, however, is not always the best or the most accurate. In addition, some studies have successfully measured startle response with fMRI measurements [70]. Ultimately, the more measurements that are used while considering the specificity of each measure, the more information can be gained about the neural processes underlying fear conditioning. This information could result in more insight into sex differences and the impact of gonadal hormones in PTSD.

## CONCLUSIONS

In conclusion, gonadal hormones have been investigated using translational models of fear conditioning with varying results. Estradiol seems to have the most apparent impact with low estradiol being associated with extinction recall deficits overall. Other effects of ovarian hormones have more sparse and less consistent results but considering levels of hormones seems to have an impact on the ability of FPS to differentiate between individuals with and without PTSD diagnoses. This underscores the importance of accounting for cycle phase or hormone levels when investigating fear conditioning processes and particularly when considering preclinical models of PTSD. In addition, we have shown that different measures, specifically SCR and FPS appear to measure different and only sometimes parallel processes during fear conditioning and studies that are investigating these processes should consider the specific measurement in both study design, conclusions, and interpretations. Further research on the influence of gonadal hormones on fear conditioning processes has the potential to illuminate potential sources of risk for and resilience to PTSD. Furthermore, a better understanding of hormonal influences on processes underlying PTSD symptomology could point to potential avenues for prevention and improved treatment to reduce the suffering experienced by those who have been exposed to traumatic events.

## Data Availability

Data sharing not applicable to this article as no datasets were generated or analyzed during the current study.

## ABBREVIATIONS

PTSD: post-traumatic stress disorder
US: unconditioned stimulus
CS+: stimulus conditioned with an aversive/danger signa
CS−: stimulus conditioned with safety signal
SCR: skin conductance response
FPS: fear-potentiated startle
